# A quantitative comparison of urine centrifugation and filtration for the isolation and analysis of urinary nucleic acid biomarkers

**DOI:** 10.1038/s41598-024-54239-x

**Published:** 2024-05-13

**Authors:** Liz-Audrey Kounatse Djomnang, Carol Li, Omary Mzava, Alexandre Pellan Cheng, Adrienne Chang, Joan Sesing Lenz, Manikkam Suthanthiran, John R. Lee, Darshana M. Dadhania, Iwijn De Vlaminck

**Affiliations:** 1 Nancy E. and Peter C. Meinig School of Biomedical Engineering, Cornell University, Ithaca, NY 14850 USA; 2 Division of Nephrology and Hypertension, Department of Medicine, New York-Presbyterian Hospital–Weill Cornell Medical Center, New York, NY 10065 USA

**Keywords:** Computational biology and bioinformatics, Systems biology, Biomarkers, Diseases, Health care, Medical research, Molecular medicine, Nephrology, Engineering, Biological techniques, Bioinformatics, Genomic analysis, Isolation, separation and purification, Sequencing

## Abstract

Urine is a rich source of nucleic acid biomarkers including cell-free DNA (cfDNA) and RNA for monitoring the health of kidney allografts. In this study, we aimed to evaluate whether urine filtration can serve as an alternative to the commonly used method of centrifugation to collect urinary fluid and cell pellets for isolating cfDNA and cellular messenger RNA (mRNA). We collected urine specimens from kidney allograft recipients and obtained the urine supernatant and cell pellet from each specimen using both filtration and centrifugation for paired analyses. We performed DNA sequencing to characterize the origin and properties of cfDNA, as well as quantitative PCR of mRNAs extracted from cell fractions. Our results showed that the biophysical properties of cfDNA, the microbial DNA content, and the tissues of origin of cfDNA were comparable between samples processed using filtration and centrifugation method. Similarly, mRNA quality and quantity obtained using both methods met our criteria for downstream application and the Ct values for each mRNA were comparable between the two techniques.The Ct values demonstrated a high degree of correlation. These findings suggest that urine filtration is a viable alternative to urine centrifugation for isolation of nucleic acid biomarkers from urine specimens.

## Introduction

Urine is a widely used biofluid for monitoring kidney allograft recipients for signs of rejection and infection. Urine cell-free DNA (cfDNA) was shown to be an informative bio-analyte to monitor for urinary tract infection and rejection^1–4^, and urinary cell messenger RNA (mRNA) profiles are diagnostic and prognostic of BK virus nephropathy and T-cell mediated rejection of kidney allografts^5–9^.

At the present time, centrifugation is the established standard method used to separate the urine supernatant and urinary cells from urine to profile cfDNA and cellular mRNA. However, this method requires specialized equipment, and expedited processing time. Membrane filtration, on the other hand, may offer a faster, simpler alternative for separating urine into its fluid and solid components that is accessible to all. In this study, we aimed to evaluate the utility of urine filtration as an alternative to centrifugation for isolating bioanalytes from urine specimens. We collected 28 urine specimens in two equal aliquots from 16 kidney transplant patients, isolated cfDNA from supernatants and mRNA from cell fractions, and performed cell-free DNA sequencing and RT-qPCR to establish the similarities and differences in cfDNA and mRNA profiles obtained through centrifugation and filtration.

We found that the biophysical properties, microbial DNA content, and tissues of origin of cfDNA in urine supernatants were comparable between samples processed through filtration and centrifugation. In addition, we found that mRNA profiles obtained from cell fractions using centrifugation and filtration resulted in similar Ct values and demonstrated a high degree of correlation.

Our study provides early evidence that urine filtration may be used as a routine method in the field of kidney transplant health monitoring and supports further exploration of its potential applications in other areas of medical research.

## Results

### cfDNA in urine fluid collected by centrifugation vs. filtration

**Figure 1:**
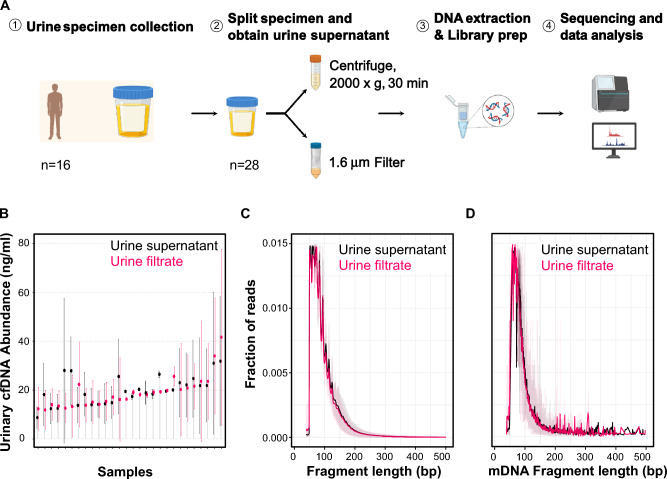
Study design and cfDNA biophysical properties. (**A**) Sample collection and processing overview; Individual urine specimens are collected and then split for processing by centrifugation or filtering. (**B**) Measurement of cfDNA in samples processed by centrifugation and filtering. Average total cfDNA abundance in samples following cfDNA extraction. (**C**) Nuclear cfDNA fragmentation profile. D. Mitochondrial cfDNA fragmentation profile.

We first set out to compare the characteristics of cell-free DNA obtained by centrifugation and filtration. Urinary cell-free DNA (cfDNA) can be used to monitor urinary tract infections (UTIs) and immune rejection after kidney transplantation^1,2,10^. cfDNA diagnostic methods rely on the total amount of cfDNA, the biophysical properties of the isolated cfDNA, the burden and properties of microbial urinary cfDNA, and the tissues of origin of host-specific DNA. Therefore, we integrated these readouts in our study design. We collected 28 urine specimens from 16 kidney transplant patients and divided each specimen into two equal portions (Figs. 1A and 3A). The first portion was processed using centrifugation to isolate the urine supernatant, while the second portion was processed using the Weill Cornell Hybrid Protocol (“Material and methods”).

We began by evaluating the total amount of urinary cfDNA obtained after centrifugation and filtration. To ensure consistency, we maintained constant urine input and elution volumes for DNA isolation for paired filtrate and supernatant samples (“Material and methods”). Our results showed that the method of urine collection did not have a significant impact on the yield of cfDNA, with average yields of 19.95 ng/ml and 18.86 ng/ml of cfDNA in the supernatants and filtrates respectively (Wilcoxon sum rank test, p-value = 0.73, Fig. 1B). For a small number of samples, the cfDNA biomass measurements were discordant, which we attribute to experimental variability or batch effect.

We next implemented cfDNA sequencing to assess the DNA fragmentation profile, tissues of origin, and composition of the urinary microbiome. We implemented Sample-Intrinsic microbial DNA Found by Tagging and sequencing^3^ (SIFT-seq), a method for metagenomic cfDNA sequencing that is robust against environmental contamination (“Material and methods”). SIFT-seq relies on the tagging and recovery of sample intrinsic cfDNA by bisulfite conversion.

We used paired-end read mapping to obtain the cfDNA fragment length profiles. The fragmentation profiles of cfDNA obtained from urine filtrate and supernatant were similar, with an average read length of 70 bp. The range of fragment lengths was highly consistent for both methods, for nuclear cfDNA (Fig. 1C) and mitochondrial cfDNA (Fig. 1D). This analysis indicates that the method of urine preprocessing does not significantly affect the fragmentation profile of cfDNA.

**Figure 2:**
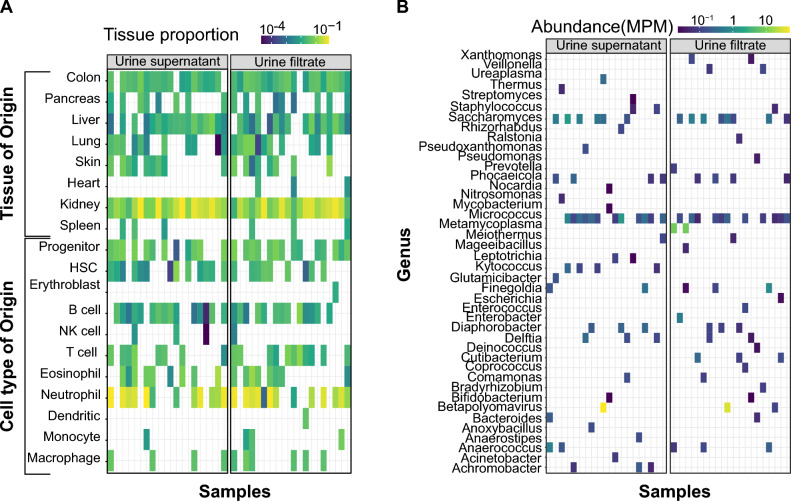
Cell-free DNA composition analysis. (**A**) Heatmap of the most tissues and cells of origin of cfDNA obtained after centrifugation and filtration. (**B**) Heatmap of the abundance of microbial cfDNA in urine obtained after filtration and centrifugation. Each column in panels A and B represents a sample, and order of the samples is the same for the supernatant and filtrate sections. R studio version 4.0.5 was used to generate all heatmaps.

We next quantified the tissues and cell type of origin of host-specific cfDNA by evaluating the methylation patterns of DNA fragments^3^. Each differentiated human cell type has a unique methylation pattern, characterized by the presence of a methyl group at the fifth carbon of cytosine in CpG regions on DNA strands^11^. By analyzing the methylation pattern of DNA fragments, it is possible to determine the tissue-of-origin of those fragments^2^. We performed a tissues-of-origin analysis using a method described by Cheng et al.^2^ Our analysis showed that the tissue-specific DNA abundances were similar in the urine filtrate and supernatant samples (p-value = 0.89, Wilcox test, Fig. 2A). This indicates that the method of urine preprocessing does not have a significant impact on the tissues-of-origin of host-specific cfDNA. In all specimens independently of the processing method, cfDNA was predominately derived from kidney cells and neutrophils.

Last, we quantified microbial cfDNA in urine filtrates and supernatants in terms of abundance, species richness, and evenness. We found that the microbial profiles in paired samples were highly similar (Figs. 2B, S1A). The abundance of different genera was consistent between paired samples (Wilcoxon sum rank test p-value = 0.42). We also evaluated the community richness and evenness of paired samples using the Simpson diversity index and found that the microbial diversity was consistent between filtrate and supernatant samples (p-value = 0.06, see supplement Fig. S1B).

### mRNA profiling of urinary cells collected by centrifugation vs. filtration

**Figure 3:**
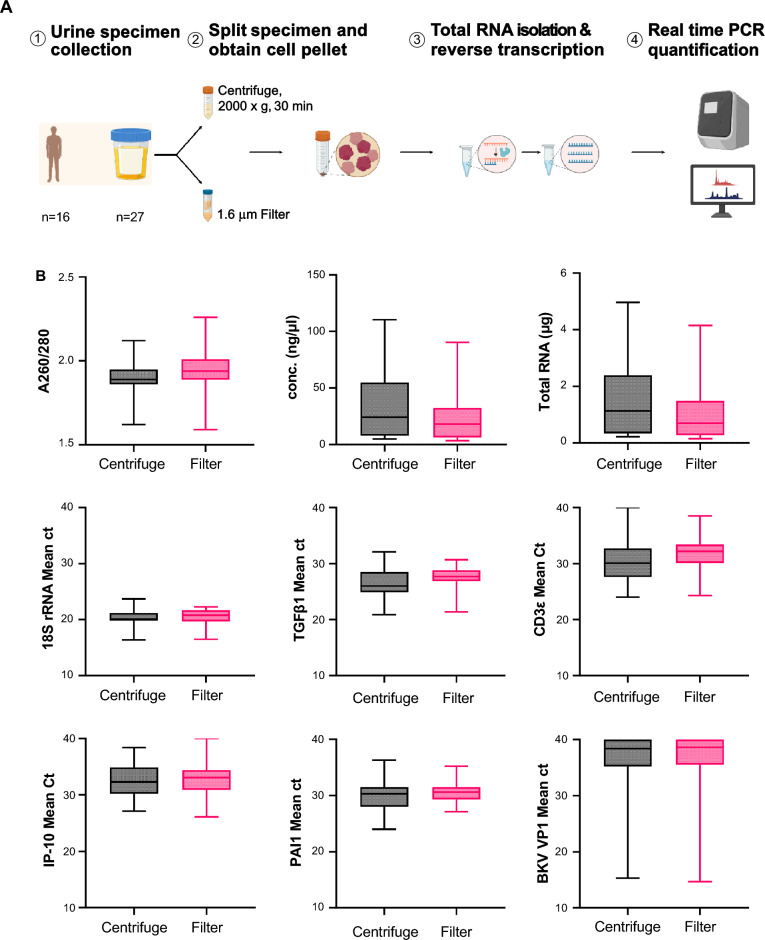
(**A**) Process overview shows the steps associated with total RNA profiling. (**B**) Box and whisker plots demonstrate the minimum, 25th, 50th, 75th quartiles, and the maximum for mRNA concentration(ng/μl), A260/280 ratio, and the total RNA (μg), and the Ct values of 18S rRNA, TGFb1, CDε IP-10, PAI-1 and BKV VP1 mRNA transcripts.

Our initial investigations identifying urine mRNA profiles as noninvasive diagnostic and prognostic biomarkers of rejection and BKVN^12^ utilized the traditional centrifugation method for collection of the urine cell fraction. More recently, we reported a novel filtration protocol that eliminated the centrifugation step for urine processing and could be used to measure the three-gene signature diagnostic of T-cell mediate rejection of kidney allograft and BKV VP1 mRNA levels, diagnostic of BKV associated nephropathy^13^. In this study, we performed a direct comparison of the urine mRNA profiles obtained from centrifugation and filtration method. We measured the mRNA yield, quality, and profiles obtained from 27 urine specimens collected as part of a monitoring study for kidney transplant patients to detect infectious complications. For each specimen, equal volumes of urine were processed simultaneously using both centrifugation and filtration (Fig. 3A). The mean starting volume of urine was 26 ± 15 ml for both techniques. The A260/A280 ratio indicating purity of total RNA was comparable (1.89 ± 0.11 for the centrifugation method versus 1.94 ± 0.13 for the filter method) as was the mean RNA concentration (34.1 ± 32.3 ng/μl for the centrifugation method and 23.7 ± 23.8 ng/µl for the filtration method, Fig. 3B). The total RNA yield was slightly higher for the centrifugation method (1.14 ± 1.42 μg) compared to the filtration method (0.70 ± 0.08 μg). All paired samples processed using both methods met our established criteria for adequate urinary cell mRNA profiling, with TGFβ1 mRNA copies > 100 copies/μg of total RNA and 18S rRNA copies > 18.5 × 10^7^ copies/µg^12^.

We used preamplification enhanced real-time quantitative PCR (RT-qPCR) to quantify the abundance of mRNA transcripts using a standard curve method as previously described^7,12^. We observed a high degree of correlation between the cycle threshold (Ct) values for the mRNA transcripts obtained from the centrifugation method versus the filtration method. As shown in Table 1, Pearson’s correlation coefficient was 0.73 for 18S rRNA, 0.65 for TGFb1, 0.50 for CD3e, 0.65 for IP-10, 0.74 for PAI-1, and 0.94 for BKV VP1. A comparison of the median Ct values for each of the mRNA transcripts is shown in Fig. 3B. The median Ct for 18S rRNA copies was 20.1 for centrifugation vs. 20.8 for filtration (p = 0.1), for TGFβ1 copies was 26 vs. 27.7 (p = 0.05), for CD3ε copies was 30.1 vs. 32.2 (p = 0.09), for IP10 copies were 32.3 vs. 33.1 (p = 0.61), and for PAI-1 copies was 30.3 vs. 30.6 (p = 0.12). These results support the use of filtration to isolate mRNA from urinary cells as an alternative to centrifugation, as the method of urine preprocessing did not significantly affect the yield, quantity, and profile of mRNA transcripts. Additionally, we calculated the 3-gene T-cell mediated rejection (TCMR) diagnostic signature using the mRNA obtained from the centrifugation method versus the filtration method. The mean 3-gene signature value was − 1.91 ± 1.40 for the centrifugation vs. − 2.27 ± 1.33 for the filtration method (p = 0.19).

**Table 1:** Pearson’s correlation of mRNA transcripts between centrifugation and filtration methods.

mRNA	r	95% confidence interval	Adj. P-value
18S rRNA	0.73	0.4896 to 0.8706	0.0006
TGFβ1	0.64	0.3509 to 0.8232	0.0009
CD3ε	0.50	0.1458 to 0.7384	0.0164
IP10	0.65	0.3570 to 0.8254	0.0009
PAI1	0.74	0.5047 to 0.8754	0.0006
BKV VP1	0.94	0.8695 to 0.9722	0.0006

## Discussion

Our previous investigations have validated the use of urine as a useful analyte for monitoring episodes of rejection and infection in kidney allograft recipients^6–9^. To facilitate adoption of urine biomarkers, we recently developed a filter-based method for ease of collecting urine supernatant and cell fractions. In this study, we performed our first direct head-to-head comparison of urine cfDNA and urinary cell mRNA quantity and integrity obtained using filtration versus centrifugation. We demonstrate that the molecular biomarkers measured using assays developed in our laboratory are comparable between the two techniques. Our results therefore show that urine filtration is a viable alternative to centrifugation for collection of urine supernatant and cell fraction for measurement of biomarkers in kidney allograft recipients.

Regarding cfDNA, we found that the total amount, the fragmentation profile, the microbial DNA content, and tissues of origin of cfDNA were all comparable between samples processed through filtration and centrifugation. This suggests that filtration does not negatively impact the diagnostic potential of cfDNA in kidney transplant monitoring for urinary tract infections and immune rejection.

When comparing mRNA profiles from cellular pellets, we observed a high degree of correlation between the filtration and centrifugation methods. Although the total RNA yield was higher for the centrifugation method, the purity of the RNA was similar between the two methods. Most importantly, we found that the paired Ct values in the RT-qPCR assay were similar between the two techniques. Moreover, 100% of the samples processed using the filtration method demonstrated adequate quantity of mRNA (TGFb1 copies > 100 per μg total RNA and 18S rRNA copies > 18.5 × 10^7^ copies per μg of total RNA). It is important to note that these measurements were obtained in patients who were within the first two months post kidney transplant surgery and had stable graft function and therefore are expected to have minimal cellular traffic within the kidney allograft. In fact, the median 3-gene TCMR diagnostic signature values were below the diagnostic threshold and similar between the centrifugation and filtration methods. We previously^13^ demonstrated that urinary mRNA profiles obtained from filtration-based methods accurately distinguish patients with T-cell mediated rejection from those with a stable function. In this manuscript, we effectively demonstrate that mRNA profiles obtained by filtration-based methods are comparable to those obtained by centrifugation-based methods.

This study has a few limitations. First, the study was not designed and did not have a sufficiently large scope in terms of the number of samples and patients included to enable, to identify, and compare biomarkers of disease as measured by the filtration and centrifugation methods. In addition, we limited the analysis to urinary cfDNA and mRNA and did not investigate other bioanalytes that are of potential interest for the monitoring of kidney transplant patients, including micro RNAs, and proteins. Future studies may explore other biomarkers and potential applications of urine filtration in other domains of medical research and clinical practice. Nonetheless, our results hold significant implications for resource-limited settings, as filtration does not necessitate specialized equipment or facilities. The simplicity and accessibility of filtration make it an attractive option for routine clinical monitoring of kidney transplant patients, particularly in settings where access to specialized equipment is limited.

## Material and methods

### Study subjects and specimens

Urinary specimens were collected from kidney transplant recipients (KTxR) enrolled in the WCM IRB protocol 20-01021269 protocol entitled “Metagenomic profiling of urinary cell-free DNA to monitor urinary tract infection after kidney transplantation”, We studied 28 paired urine specimens from 16 KTxR, 12 KTxR provided specimens at 2 different time points and 4 provided at a single time point. The study cohort included 11 (69%) male and 10 (63%) living donor transplant recipients. The mean age of the study cohort was 50 ± 11.7 years. Urinary samples were collected at 46 ± 10.9 days post-transplant, and 46% (13/28) urine specimens were collected in the presence of a ureteral stent in the transplanted kidney. At the time of specimen collection, none of the patients had symptoms of urinary tract infection. All specimens and patient participation were approved under the Institutional Division of Human Subject Protection (IRB protocol 20-01021269). Informed consent was obtained from all participants. All experiments were performed in accordance with relevant guidelines and regulations.

### Urine collection and processing

Patients were asked to provide mid-stream clean catch urine specimen. All urine specimens were either processed immediately or stored at 4 °C for up to 4 h after collection. After collecting 5 ml for urine culture using BD Vacutainer^®^, UA No Additive tubes and 5 ml for urinalysis using BD Vacutainer^®^, Urine Culture preservative tubes, we split the remaining urine into two samples of equal volume to compare the two methodologies for separating cell-free DNA and urinary cellular fractions. Our traditional centrifugation method^6,12^ used in the CTOT-04 trial was compared to our laboratory developed filtration method, Weill Cornell Hybrid Protocol (WCHP)^13^. To evaluate the performance of both methods in measuring microbial cfDNA, the urine supernatant collected after centrifugation at 2000*g* for 30 min using the in the centrifugation method was compared to the urine effluent collected after passing the thru ZRC GF™ filter using in the WCHP method. In parallel, the urinary cell mRNA obtained from cell pellet lysates in the centrifugation method were compared to the cell lysates obtained from the WCHP method. Preservative free cell-free DNA and cell lysates were stored at − 80 °C for down-stream analysis.

### cfDNA isolation, sequencing, and quantification

We extracted cfDNA from the urine fluid using QIAGEN’s QIAamp Circulating Nucleic Acid Kit (QIAGEN Cat# 55114). We followed QIAGEN’s protocol for DNA extraction in 1 ml of urine. We used Invitrogen’s Qubit dsDNA HS Assay Kit (Invitrogen Cat# Q33231) and Invitrogen Qubit 2.0 according to the manufacturer’s protocol to quantify cfDNA abundance post-isolation and to calculate the total cfDNA abundance in the urine fluid. To find the measurement error, we performed multiple cfDNA isolations. We made technical replicates of the extracts for which we obtained the Qubit values. The error was calculated as the mean total cfDNA abundance plus or minus the standard deviation for all measurements.

### cfDNA bisulfite conversion, single-stranded cfDNA isolation, and sequencing library preparation

cfDNA was extracted from urine using Norgen’s urine Cell-Free Circulating DNA Purification Midi Kit (Norgen Cat #56700). The protocol was adapted to allow bisulfite salt conversion of nucleic acids prior to extraction. Here, an aliquot of 520 µL of urine was centrifuged at 15,000 RPM for 5 min to pellet cellular and other solid debris. 500 µL of supernatant was transferred to a new 15 mL falcon tube containing 3.25 mL of ammonium bisulfite solution (Zymo Research, product #5030) and heated to 98 °C for 15 min. Samples were then kept at 54 °C for 90 min. Then, cfDNA extraction was performed using a commercially available column-based kit (Norgen Biotek, product #56700). Before cfDNA elution, 200 µL of L-Desulphonation buffer (Zymo Research, product #5030) was added to the columns for 20 min, followed by two washes with 200 µL absolute ethanol. DNA was then eluted to 32 μl according to manufacturer recommendations. A single-stranded library preparation was performed using the SRSLY Pico-Plus DNA NGS Library Preparation Base Kit (Claret Biosciences, Cat# CBS-K250B-24). Libraries were then sequenced on a Nextseq 550 Illumina sequencer with a 2 × 75 bp read length.

### Alignment to the human genome

Adapter and low-quality bases from the reads were trimmed using BBDuk^14^ and aligned to the C-to-T and G-to-A converted human genome using Bismark^15^ (Bismark-0.22.1). PCR duplicates were removed using Bismark. We estimated bisulfite conversion efficiency by quantifying the rate of C[A/T/C] methylation in human-aligned reads (using Meth Pipe V3.4.3).

### Metagenomic abundance estimation from sequencing data

The metagenomic analysis is performed as previously described^1,3,4,16^. Specific to SIFT-seq, read-level filtering of contaminants is performed by removing sequenced reads with 4 or more cytosines present or one methylated CpG dinucleotide (the latter represents unmapped, human-derived molecules). Species-level filtering based on the distribution of mapped reads is carried out by first aligning filtered and unfiltered datasets independently. Cytosine densities of mapping coordinates in both datasets are measured using custom scripts, and their distributions are compared using a Kolmogorov–Smirnov test. Significantly different filtered-unfiltered distributions are further processed (D-statistic > 0.1 and p-value < 0.01). Briefly, filtered datasets whose distribution of cytosines at mapped locations is significantly lower than unfiltered datasets have one read removed and are re-tested for differences in their distribution. If the distributions are more similar (as measured through the same criteria), it is filtered out. This process is repeated until distributions are no longer significantly different or if all reads are removed. Metagenomic abundances of filtered datasets are estimated using GRAMMy (version 1)^17^. Microbial abundance in downstream analyses was quantified as Molecules Per Million reads (MPM).

### Total RNA isolation

In the centrifugation method, urinary cell lysates were prepared by centrifugation of whole urine at 2000*g* for 30 min and harvesting the cell pellets followed by cell lysis with 350 μl of Buffer RLT (Qiagen, Cat# 79216) + β-mercaptoethanol. In the filtration method, urine was pushed through a Whatman 1.6 μm GF/A filter and urinary cell lysate was collected by pushing 700 μl of Urine RNA Buffer (Zymo Research, Cat# R1038-2-50). Total RNA was extracted from both lysates with RNeasy Mini Kit (Qiagen, Cat 74104). The purity of isolated total RNA (A260/280) and the amount of RNA were measured using the Nanodrop One spectrophotometer. Nanodrop replicates were within 0.2 for the A260/280 ratio and 1 ng/μl for the concentration.

### Reverse transcription of total RNA to cDNA

Total RNA was reversed transcribed to cDNA using TaqMan Reverse Transcription Kit (Applied Biosystems, Cat N8080234). To normalize the different total RNA yields from the samples, total RNA was converted to cDNA at a concentration of 1 μg total RNA in 100 μl volume. The reaction contained 1 × TaqMan reverse transcription buffer, 500 μM each of 4 dNTPs, 2.5 μM of Random Hexamer, 0.4 Unit/μl of RNase inhibitor, 1.25 Unit/μl of MultiScribe Reverse Transcriptase and 5.5 mM of Magnesium Chloride, and was heated at 25 °C for 10 min, 48 °C for 30 min, and 95 °C for 5 min.

### Preamplification enhanced real time PCR quantification

Each cDNA was pre-amplified with custom-designed primer pairs using Platinum^®^ Multiplex PCR Master Mix (Applied Biosystems, Cat 4464268). Each reaction contained 5.0 μl Platinum^®^ Multiplex PCR Master Mix, 3.0 μl cDNA, 1.68 μl primer mix (50 μM sense and 50 μM antisense primers for each gene), and 0.32 μl RNase/DNase free water. The reaction was heated at 95 °C for 2 min, followed by 11 cycles of 95 °C for 30 s, 60 °C for 90 s, and 72 °C for 1 min, and held for 72 °C for 10 min. The 10 μl of pre-amplified cDNA is then diluted in 290 μl TE to use in the absolute quantification of mRNAs performed on the QuantSudio™ 6 Flex Real Time PCR system (Applied Biosystems).

### Statistical analysis

All statistical methods for cfDNA analysis were performed in R version 4.0.5. Groups were compared using two-sided Wilcoxon Rank Sum and Kolmogorov–Smirnov tests. The correlation between the Ct values obtained in the quantitative PCR (qPCR) assays following total RNA extraction using the centrifugation and filtration method were compared using Pearson’s method. Boxplots with 10th, 25th, 50th, 75th and 90^th^ percentile of the RNA parameters, concentration, amount and A260/280 ratios, as well as the Ct values obtained in the quantitative PCR (qPCR) assays were created. Boxes in the boxplots indicate the 25th and 75th percentile, the band inside the box indicates the median value and the whiskers extend to 10th and the 90th percentile. The paired Ct values associated with each mRNA measurements were compared using the Wilcoxon signed-rank test, followed by a Bonferroni correction method to adjust for type I error. P values are reported for those that remained significant after Bonferroni correction and ‘ns’ is reported if the p values were not significant after applying the Bonferroni correction.

### Supplementary Information

Supplementary Figure S1.

## Data Availability

Sequencing data from human urine cfDNA is available in the database of Genotypes and Phenotypes (dbGaP), accession number phs001564.v3.p1.
