# Supplementary material for: A quantitative comparison of urine centrifugation and filtration for the isolation and analysis of urinary nucleic acid biomarkers
Source: Sci Rep. 2024 May 13;14:10872. doi: 10.1038/s41598-024-54239-x (PMC11091160; doi:10.1038/s41598-024-54239-x)
Supplement: Supplementary file 1 — Supplementary Figure S1. [file 41598_2024_54239_MOESM1_ESM.docx]

**A Quantitative Comparison of Urine Centrifugation and Filtration for the Isolation and Analysis of Urinary Nucleic Acid Biomarkers**

**Authors:** Liz-Audrey Djomnang* ^1^, Carol Li* ^2, 3, 4^, Omary Mzava ^1^, Alexandre Pellan Cheng ^1^, Adrienne Chang ^1^, Joan Lenz ^1^, Manikkam Suthanthiran  ^2, 3, 4^, John Lee ^2, 3, 4^, Darshana Dadhania* ^2, 3, 4 #^, Iwijn De Vlaminck* ^1 #^.

**Affiliations:**

**^1^** Nancy E. and Peter C. Meinig School of Biomedical Engineering, Cornell University, Ithaca, New York, 14850, USA.

**^2^** Division of Nephrology and Hypertension, Department of Medicine; Department of Transplantation Medicine, New York-Presbyterian Hospital–Weill Cornell Medical Center, New York, NY, 10065, USA

**^3^** Department of Transplantation Medicine, New York-Presbyterian Hospital–Weill Cornell Medical Center, New York, NY, 10065, USA

**^4^** Weill Cornell Medicine, New York, NY, USA

* These Authors made equal contributions

^#^ Corresponding authors

**Supplemental figures and tables**


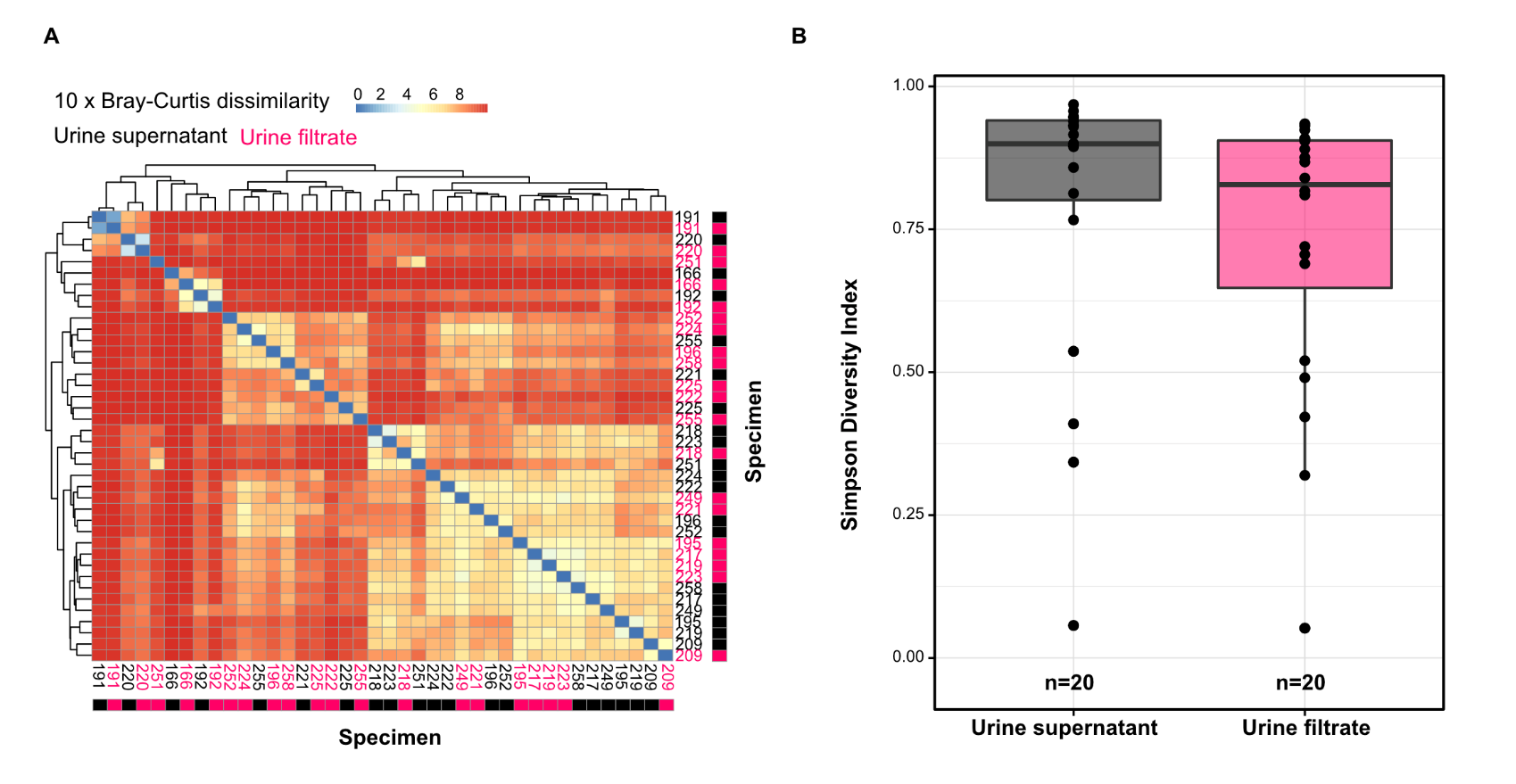


**Figure S1**: Microbial diversity. **A**. Bray-Curtis dissimilarity and unsupervised clustering; **B.** Simpson diversity
